# Retraction: Curcumin Blunts IL-6 Dependent Endothelial‐to‐Mesenchymal Transition to Alleviate Renal Allograft Fibrosis Through Autophagy Activation

**DOI:** 10.3389/fimmu.2021.818539

**Published:** 2021-11-26

**Authors:** Frontiers Editorial Office

The journal and Chief Editors retracts the 28 May 2021 article cited above.

Following publication, concerns were raised by the authors regarding the integrity of the data in the published article. Specifically, the authors have stated that the dosages of curcumin used for **Figure 7** were incorrect due to miscalculations. During the investigation, our Research Integrity team confirmed that the integrity of the figures was insufficient to support the scientific claims of the paper. The article is therefore being retracted.

This retraction was approved by the Chief Editors of Frontiers in Immunology and the Chief Executive Editor of Frontiers. The authors requested and agreed to this retraction.

